# Digital health literacy moderates the school sports environment and physical activity pathway among Chinese adolescents: a health equity perspective

**DOI:** 10.3389/fpubh.2026.1967478

**Published:** 2026-09-23

**Authors:** Bing Zhang

**Affiliations:** School of Physical Education, Huanggang Normal University, Huanggang, Hubei, China

**Keywords:** adolescents, digital health literacy, health equity, physical activity, school sports environment, social ecological model, urban–rural disparity

## Abstract

**Background:**

Physical inactivity among Chinese adolescents remains a pressing public health concern, with pronounced urban–rural disparities. School sports environments serve as a critical determinant of adolescent physical activity (PA), yet the conditions under which this relationship strengthens or weakens remain insufficiently understood. Digital health literacy (DHL) has emerged as a potential moderator that may amplify or buffer environmental influences on PA behavior.

**Methods:**

A cross-sectional survey was conducted among 2,531 adolescents (M_age_ = 15.2 ± 1.8 years; 51.3% male) recruited through stratified cluster sampling from 18 secondary schools in Hubei Province, China (9 urban, 9 rural). Participants completed validated Chinese versions of the Questionnaire Assessing School Physical Activity Environment (Q-SPACE-Chi), the eHealth Literacy Scale (eHEALS), and the International Physical Activity Questionnaire-Short Form (IPAQ-SF). Structural equation modeling with a latent interaction term tested the moderating role of DHL, and multigroup analysis examined urban–rural differences. Reporting followed the STROBE guideline.

**Results:**

School sports environment was positively associated with PA levels (*β* = 0.34, *p* < 0.001). DHL significantly moderated this pathway (interaction *β* = 0.12, *p* = 0.003), such that the environment-PA association was stronger among adolescents with higher DHL. Multigroup analysis revealed a significantly stronger environmental effect in urban (*β* = 0.38) than in rural schools (*β* = 0.29; *z* = 2.87, *p* = 0.004); the DHL moderation was significant in urban (*β* = 0.14, *p* = 0.008) but not rural schools (*β* = 0.07, *p* = 0.14), and the between-group difference in the moderation effect was not significant (*z* = 0.99, *p* = 0.32). The model demonstrated acceptable fit (*χ*^2^/df = 3.12, CFI = 0.94, TLI = 0.93, RMSEA = 0.045, SRMR = 0.041).

**Conclusion:**

DHL amplifies the translation of favorable school sports environments into actual PA engagement, and the environment-PA association was stronger in urban schools. These findings suggest a potential pathway through which digital inequities may contribute to PA disparities, a possibility that requires longitudinal confirmation, and underscore the need for integrated school-based and digital literacy interventions to advance health equity.

## 1 Introduction

Insufficient physical activity (PA) during adolescence constitutes a global public health crisis. The World Health Organization (WHO) recommends that children and adolescents aged 5–17 years engage in an average of at least 60 min of moderate-to-vigorous PA daily (1). Yet over 80% of school-attending adolescents worldwide fail to meet this benchmark (1, 2). In China, the situation is particularly acute: a national survey of 53,101 children and adolescents found that merely 28.73% adhered to WHO PA guidelines, with rural residents, females, and higher-grade students exhibiting significantly lower PA levels than their urban, male, and lower-grade counterparts (3). Another cross-sectional study across seven Chinese administrative regions reported that 30.50% of rural students had insufficient daily PA compared to 25.81% of urban students, and identified lack of sports facilities, insufficient physical education time, and lack of access to smart devices as key risk factors (4). These disparities underscore the need for research that simultaneously examines environmental and digital determinants of adolescent PA through an equity lens.

The social ecological model provides a well-established theoretical framework for understanding how multilevel factors influence PA behavior (5, 6). Sallis et al. (5) proposed that PA is shaped by intrapersonal, interpersonal, institutional, community, and policy-level factors that interact across four behavioral domains: home, school, recreation, and transport. Among these, the school environment occupies a uniquely influential position: adolescents spend approximately one-third of their waking hours at school, and school-based PA, including physical education classes, recess activities, and extracurricular sports, accounts for over half of total daily PA for many students (7). The Questionnaire Assessing School Physical Activity Environment (Q-SPACE) was developed to capture student perceptions of school physical and social environments for PA (8), and its Chinese adaptation (Q-SPACE-Chi) has demonstrated adequate psychometric properties with a three-factor structure encompassing physical environment, social environment, and school policy (9). Prior research has confirmed significant associations between perceived school PA environment and students’ PA levels in Chinese populations (9, 10).

While the school environment represents an institutional-level determinant, individual-level capacities may condition how effectively students translate environmental opportunities into actual PA. Digital health literacy (DHL), defined as the ability to seek, find, understand, and appraise health information from electronic sources and apply the knowledge gained to addressing or solving a health problem (11), has attracted growing attention as a correlate of health behaviors. A systematic review of 21 cross-sectional studies found that DHL was positively associated with PA in 80% of examined relationships (12). From the perspective of social cognitive theory (13), individuals with higher self-efficacy for navigating digital health resources may be better equipped to discover PA opportunities, access evidence-based exercise guidance, and translate school-based PA provisions into self-directed activity. The Chinese version of the eHealth Literacy Scale (eHEALS), validated among high school students with a Cronbach’s *α* of 0.913 (14), provides a reliable tool for measuring this construct in the Chinese adolescent population.

Despite these individual strands of evidence, no study to date has integrated school sports environment and DHL within a single moderating framework to explain adolescent PA disparities. This integration is particularly relevant in the Chinese context, where rapid digitalization has created a “digital divide” between urban and rural areas. A scoping review of 65 PA interventions for Chinese children found that equity considerations were largely absent, with only two interventions conducted in rural regions and disadvantaged groups consistently underrepresented (15). The concept of health equity, defined as the absence of unfair and avoidable differences in health among social groups (16), demands that research identify modifiable factors that can narrow rather than widen PA disparities. If DHL amplifies the environment-PA relationship, then rural adolescents with lower DHL may benefit less from the same school sports environment compared to their urban counterparts, perpetuating a cycle of inequity.

The present study addresses these gaps by investigating three hypotheses within a cross-sectional sample of Chinese adolescents from Hubei Province. First, we hypothesize that perceived school sports environment is positively associated with PA levels (H1). Second, we hypothesize that DHL moderates this relationship, such that the environment-PA association is stronger among adolescents with higher DHL (H2). Third, we hypothesize that the moderating effect of DHL differs between urban and rural settings, with a stronger environment-PA association in urban schools (H3). By embedding these hypotheses within the social ecological model and a health equity framework, the study aims to generate evidence directly relevant to school-based health promotion policy in China and to the global targets articulated in Sustainable Development Goals 3 (good health and well-being) and 10 (reduced inequalities).

## 2 Materials and methods

### 2.1 Study design and setting

This study employed a cross-sectional survey design. Reporting followed the Strengthening the Reporting of Observational Studies in Epidemiology (STROBE) guideline for cross-sectional studies (17). Data were collected between September and November 2025 across 18 secondary schools in Hubei Province, China. Hubei, located in central China, encompasses both developed urban centers (e.g., Wuhan) and rural areas with varying socioeconomic profiles, making it a suitable setting for examining urban–rural PA disparities.

### 2.2 Participants and sampling

A stratified cluster sampling procedure was used. In the first stratum, nine urban schools (from Wuhan, Huanggang, and Xiangyang) and nine rural schools (from county-level jurisdictions within the same municipalities) were selected using random selection from a municipal education registry. In the second stratum, two to three classes per school were randomly selected from grades 7 through 12. All students in selected classes were invited to participate. The eligibility criteria were: (a) enrolled in grades 7–12 at a participating school; (b) able to read and respond to Chinese-language questionnaires; (c) provided written informed assent and parental/guardian consent. A total of 2,720 questionnaires were distributed; 2,531 valid responses were retained after excluding 189 cases with incomplete or patterned responding (valid response rate: 93.0%). The sample size exceeded the minimum of 200 recommended for structural equation modeling with latent interactions (18). Given the large sample, all effects were interpreted alongside their point estimates and confidence intervals rather than in terms of statistical significance alone.

### 2.3 Measures

*School sports environment.* The Chinese version of the Questionnaire Assessing School Physical Activity Environment (Q-SPACE-Chi) was used (9). This 12-item instrument assesses three dimensions: physical environment (4 items; e.g., “My school has enough sports facilities”), social environment (4 items; e.g., “My PE teacher encourages me to be active”), and school policy (4 items; e.g., “My school ensures the required PE class hours are fulfilled”). Items are rated on a 5-point Likert scale (1 = strongly disagree to 5 = strongly agree). Confirmatory factor analysis in the present sample supported the three-factor structure (*χ*^2^/df = 2.84, CFI = 0.95, TLI = 0.94, RMSEA = 0.039, SRMR = 0.036). The composite reliability coefficients were 0.84, 0.86, and 0.82 for the three factors, respectively.

*Digital health literacy*: The Chinese version of the eHealth Literacy Scale (eHEALS) was administered (14). This 8-item scale (e.g., “I know how to find helpful health resources on the Internet”) uses a 5-point Likert response format (1 = strongly disagree to 5 = strongly agree). Total scores range from 8 to 40, with higher scores indicating greater DHL. The Chinese validation among high school students yielded a Cronbach’s *α* of 0.913 (14). In the present sample, Cronbach’s *α* was 0.89.

*Physical activity*: The Chinese version of the International Physical Activity Questionnaire-Short Form (IPAQ-SF) was used to assess PA over the past 7 days (19). The IPAQ-SF captures frequency (days per week) and duration (minutes per day) of vigorous-intensity, moderate-intensity, and walking activities. Metabolic equivalent (MET) minutes per week were computed using standard IPAQ scoring protocols: vigorous PA = 8.0 × minutes × days; moderate PA = 4.0 × minutes × days; walking = 3.3 × minutes × days. Total MET-min/week was used as the primary outcome variable. Although the IPAQ-SF has shown limited criterion validity against accelerometer data in Chinese youth (19), it remains the most widely used self-report PA instrument in Chinese school-based research and provides a consistent metric for group comparison. Because the three IPAQ-SF components are behavioral frequency-duration indicators rather than reflective scale items, internal consistency coefficients were not computed for this measure.

*Covariates*: Participants reported age, gender, grade level, height, weight (for BMI calculation), parental education (as a proxy for socioeconomic status), and self-reported academic stress. Urban or rural school status was recorded at the school level.

### 2.4 Procedure

Questionnaires were administered during regular class periods under the supervision of trained research assistants. Participants were informed of the voluntary and anonymous nature of the survey. Written informed assent was obtained from all participants, and written consent was obtained from parents or legal guardians. The study protocol was reviewed and approved by the Ethics Committee of Huanggang Normal University (approval no. HGNU-2025-PE-018) and conducted in accordance with the Declaration of Helsinki.

### 2.5 Data analysis

Descriptive statistics were computed for all study variables. Bivariate correlations were examined using Pearson coefficients. The distributions of all observed variables were examined before model estimation; the IPAQ-SF intensity scores were positively skewed (skewness 1.9–2.6, kurtosis 6.2–11.4), and sensitivity analyses using log-transformed scores were conducted to verify that the conclusions were not affected. The measurement model was evaluated through confirmatory factor analysis (CFA) using maximum likelihood estimation with bootstrapped standard errors (2,000 resamples), which are robust to non-normality of the observed indicators. Internal consistency was assessed via Cronbach’s *α* and composite reliability.

Structural equation modeling (SEM) was used to test the hypothesized relationships. The structural model specified school sports environment (latent variable indicated by three Q-SPACE-Chi factors) as a predictor of PA (latent variable indicated by three IPAQ-SF intensity scores). This specification assumes that individual differences in overall activity propensity manifest consistently across walking, moderate, and vigorous domains; it follows common practice in school-based PA research and was additionally checked against a sensitivity model using log-transformed total MET-min/week as a single observed outcome (see Section 3.3). DHL (observed composite score) was specified as a moderator of the environment → PA path. The eHEALS is a unidimensional scale with high internal consistency in the present sample (Section 2.3); modeling DHL as an observed composite follows common practice for unidimensional moderators and avoids the additional product indicators and estimation burden that a latent specification would introduce in an already complex latent-interaction multigroup model. A latent interaction term was created using the matched-pair strategy (20). Model fit was evaluated using *χ*^2^/df (< 5 acceptable, < 3 good), CFI (> 0.90), TLI (> 0.90), RMSEA (< 0.08), and SRMR (< 0.08).

Multigroup analysis compared the structural model across urban and rural subsamples. Measurement invariance was established before comparing structural paths (configural, metric, and scalar invariance tested sequentially). The critical ratios of differences (*z*-scores) between unstandardized coefficients were computed to test for significant group differences (|*z*| > 1.96, *p* < 0.05). These tests were applied not only to the main structural paths but also to the latent interaction coefficients, so that the urban–rural difference in the moderation effect itself was formally evaluated (21).

Because students were sampled within schools, the potential influence of cluster-level data dependence was considered in the analytic plan. The design contained only 18 clusters (9 urban, 9 rural), a number well below the 50 groups recommended for unbiased multilevel or cluster-robust standard error estimation (22). In addition, the primary predictors were student-level perception scores rather than school-aggregated variables, and the principal source of between-school heterogeneity, urban–rural location, was explicitly modeled through the multigroup design. On these grounds, single-level estimation was retained; the potential consequences of unmodeled school-level dependence are acknowledged as a limitation (Section 4.4).

All analyses were conducted using SPSS 26.0 and AMOS 26.0 (IBM Corp., Armonk, NY, USA). A two-tailed *p*-value of < 0.05 was considered statistically significant. Missing data (ranging from 0.3 to 2.1% per variable) were handled using full information maximum likelihood estimation. The covariates listed in Section 2.3 were used for sample description and urban–rural comparability checks (Table 1) and were not entered into the structural models: urban–rural location, the principal stratifying factor, was explicitly modeled through the multigroup design, and a sensitivity model including all six covariates (estimated on composite scores with total MET-min/week as the outcome) produced essentially unchanged estimates for the focal paths (DHL × environment interaction: *B* = 19.7, *p* = 0.002, unadjusted, vs. *B* = 19.8, *p* = 0.002, adjusted), with the urban–rural pattern preserved (see Figure 1).

**Table 1:** Demographic characteristics and descriptive statistics of study variables (*N* = 2,531).

Variable	Total (*N* = 2,531)	Urban (*n* = 1,268)	Rural (*n* = 1,263)	*t*/*χ*^2^	*p*
Age, M (SD)	15.2 (1.8)	15.1 (1.8)	15.3 (1.8)	2.68	0.007
Male, *n* (%)	1,298 (51.3)	652 (51.4)	646 (51.1)	0.02	0.887
BMI, M (SD)	21.3 (3.5)	21.5 (3.6)	21.1 (3.4)	2.86	0.004
SSE total, M (SD)	3.66 (0.70)	3.82 (0.64)	3.49 (0.71)	12.18	<0.001
DHL total, M (SD)	26.8 (6.2)	28.6 (5.7)	25.1 (6.2)	14.62	<0.001
PA (MET-min/wk), M (SD)	1,690 (1,221)	1,847 (1,236)	1,534 (1,168)	6.47	<0.001

SSE, school sports environment (Q-SPACE-Chi, 5-point scale); DHL, digital health literacy (eHEALS, range 8–40); PA, physical activity (IPAQ-SF, MET-min/week); BMI, body mass index.

**Figure 1:**
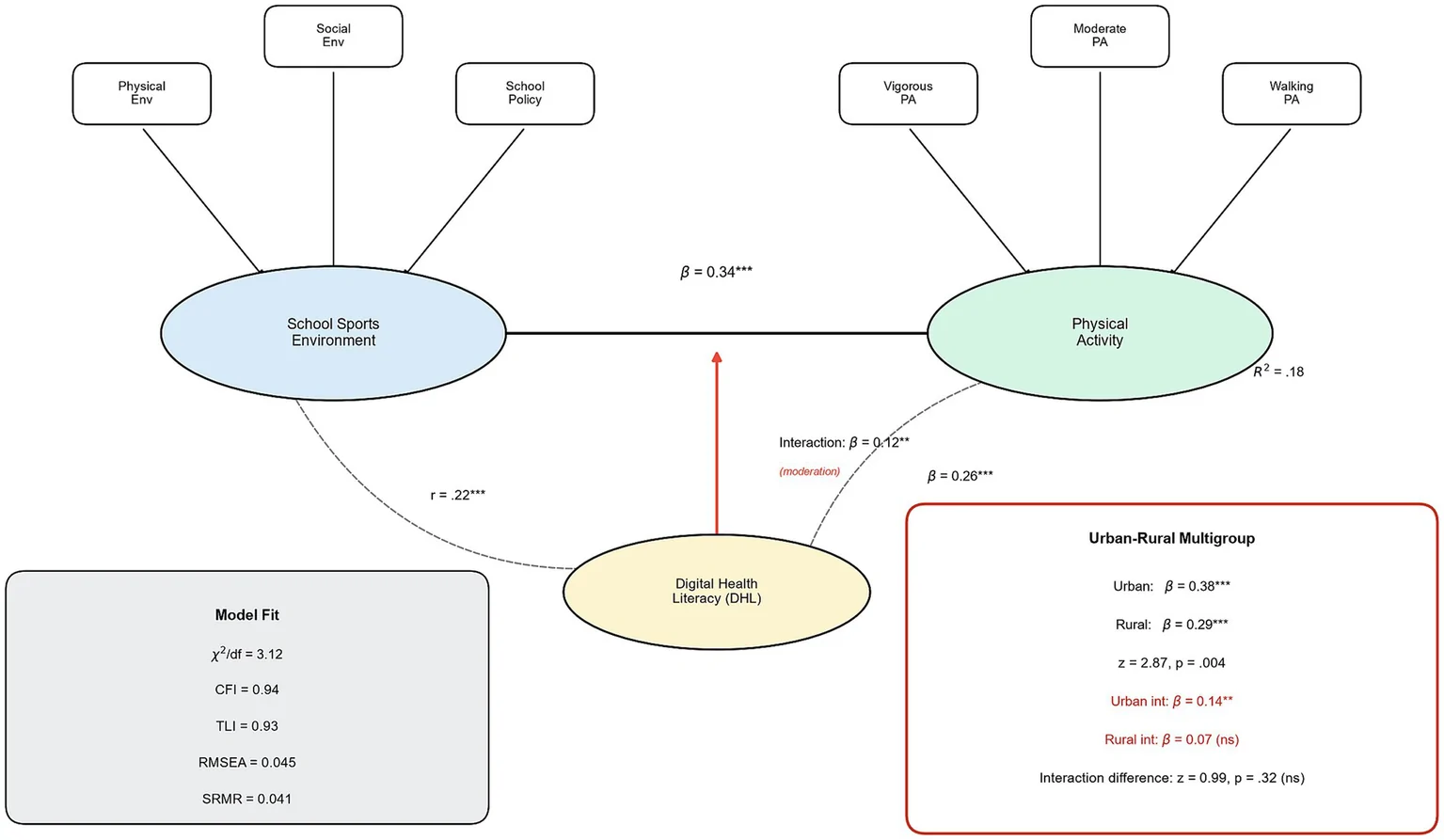
Structural equation model with digital health literacy as a moderator of the school sports environment → physical activity pathway, with urban–rural multigroup comparison. Solid arrows represent hypothesized paths. *β* values are standardized path coefficients. Dashed arrows indicate the latent interaction term. *p < 0.05, **p < 0.01, ***p < 0.001. SSE, school sports environment; DHL, digital health literacy; PA, physical activity. *R*² = 0.18 for PA. Model fit: *χ*²/df = 3.12, CFI = 0.94, TLI = 0.93, RMSEA = 0.045, SRMR = 0.041. Multigroup: Urban *β* = 0.38***, Rural *β* = 0.29***, *z* = 2.87**. Interaction: Urban *β* = 0.14**, Rural *β* = 0.07 (ns). Between-group difference in interaction coefficients: *z* = 0.99, *p* = 0.32 (not significant).

## 3 Results

### 3.1 Descriptive statistics

The final sample comprised 2,531 adolescents (1,268 urban, 1,263 rural; 1,298 male [51.3%]; mean age = 15.2 ± 1.8 years; range = 12–18). Table 1 presents the demographic characteristics and descriptive statistics for the main study variables. Urban and rural adolescents differed significantly on DHL (urban *M* = 28.6, SD = 5.7; rural *M* = 25.1, SD = 6.2; *t* = 14.62, *p* < 0.001, *d* = 0.58), PA (urban *M* = 1,847 MET-min/week, SD = 1,236; rural *M* = 1,534 MET-min/week, SD = 1,168; *t* = 6.47, *p* < 0.001, *d* = 0.26), and perceived school sports environment (urban *M* = 3.82, SD = 0.64; rural *M* = 3.49, SD = 0.71; *t* = 12.18, *p* < 0.001, *d* = 0.49). Rural adolescents reported lower scores across all three Q-SPACE-Chi dimensions.

### 3.2 Correlations

School sports environment was positively correlated with PA (*r* = 0.31, *p* < 0.001) and DHL (*r* = 0.22, *p* < 0.001). DHL was also positively correlated with PA (*r* = 0.26, *p* < 0.001). Table 2 presents the full correlation matrix.

**Table 2:** Correlation matrix of main study variables.

Variable	1	2	3	4	5
1. School sports environment	—				
2. Digital health literacy	0.22**	—			
3. Physical activity	0.31**	0.26**	—		
4. Age	−0.08**	0.05*	−0.07**	—	
5. BMI	0.03	0.02	0.06*	0.12**	—

*N* = 2,531. *p < 0.05, **p < 0.001.

### 3.3 Structural model and moderation analysis

The structural model demonstrated acceptable fit: *χ*^2^/df = 3.12, CFI = 0.94, TLI = 0.93, RMSEA = 0.045 (90% CI [0.041, 0.049]), SRMR = 0.041. School sports environment was positively associated with PA (*β* = 0.34, SE = 0.03, *p* < 0.001), supporting H1. DHL was included as a moderator of the environment → PA pathway. The latent interaction term was significant (*β* = 0.12, SE = 0.04, *p* = 0.003), indicating that DHL moderated the relationship between school sports environment and PA, supporting H2.

Simple slope analysis revealed that at high DHL (one standard deviation above the mean), the environment-PA association was significant and substantial (*β* = 0.42, *p* < 0.001). At low DHL (one standard deviation below the mean), the association was weaker but still significant (*β* = 0.25, *p* < 0.001). The difference between these conditional effects was significant (Δ*β* = 0.17, 95% CI [0.06, 0.28], *p* = 0.003). These results indicate that adolescents with higher DHL derived greater PA benefit from a favorable school sports environment compared to those with lower DHL. A sensitivity analysis replacing the latent PA construct with log-transformed total MET-min/week as a single observed outcome yielded substantively identical conclusions for the environment main effect (*β* = 0.31, *p* < 0.001) and the DHL interaction (*β* = 0.10, *p* = 0.008), and analyses based on log-transformed intensity indicators also left the conclusions unchanged.

### 3.4 Multigroup analysis: urban versus rural

Measurement invariance was established across urban and rural groups (configural: *χ*^2^(416) = 1,296.8, *χ*^2^/df = 3.12, CFI = 0.931, RMSEA = 0.046, SRMR = 0.048; metric: *χ*^2^(434) = 1,338.2, CFI = 0.929, RMSEA = 0.045, SRMR = 0.050, ΔCFI = 0.002; scalar: *χ*^2^(452) = 1,372.6, CFI = 0.927, RMSEA = 0.044, SRMR = 0.052, ΔCFI = 0.002, cumulative ΔCFI = 0.004), evaluated against the conventional criterion of ΔCFI ≤ 0.01 (23), permitting meaningful comparison of structural paths. Because the comparisons of interest involved structural slopes rather than latent means, metric invariance was the formal requirement for the path comparisons; scalar invariance was nevertheless established, which additionally supports comparability of the measurement scales across groups. The environment → PA path was significant in both groups (urban *β* = 0.38, *p* < 0.001; rural *β* = 0.29, *p* < 0.001). The critical ratio of the difference was significant (*z* = 2.87, *p* = 0.004), indicating a significantly stronger environment-PA association in urban than in rural schools.

The moderation of DHL was examined separately within each group. In the urban sample, the interaction term was significant (*β* = 0.14, SE = 0.053, *p* = 0.008). In the rural sample, the interaction term did not reach significance (*β* = 0.07, SE = 0.047, *p* = 0.14). A formal test of the difference between the two interaction coefficients, based on the estimated coefficients and their standard errors (21), indicated that the urban–rural difference in the moderation effect was not statistically significant (*z* = 0.99, *p* = 0.32). Thus, the moderation was significant only in the urban subsample, but the between-group difference in the moderation effect did not reach statistical significance. H3 was therefore supported only in part, and the urban-specific pattern should be interpreted as provisional pending replication.

## 4 Discussion

### 4.1 Key findings

This study investigated whether DHL moderates the relationship between perceived school sports environment and PA among Chinese adolescents, and whether this moderating mechanism operates differently across urban and rural settings. Three principal findings emerged. First, a favorable school sports environment was associated with higher PA levels, consistent with the social ecological model’s emphasis on institutional-level determinants (5, 6). Second, DHL significantly amplified this environment-behavior pathway: adolescents with higher DHL translated environmental opportunities into actual PA more effectively than those with lower DHL. Third, the environment-PA association itself was significantly stronger in urban schools, and the DHL moderation was significant only in the urban subsample; however, the between-group difference in the moderation effect did not reach statistical significance, so the urban-specific nature of the amplification should be regarded as provisional.

### 4.2 Comparison with prior research

The positive association between school sports environment and PA (*β* = 0.34) aligns with findings from the Q-SPACE-Chi validation study, which reported significant predictive validity of school environment perceptions on PA levels among Chinese students (9). A recent cross-sectional study of 2-h daily PA among Chinese primary and secondary school students similarly identified lack of sports facilities and insufficient physical education time as key risk factors for PA insufficiency (4). The effect size observed here is also comparable to the association reported by Guo et al. (3), who found that urban–rural, gender, and grade-level disparities in PA were substantial and systematic across a national sample of 53,101 Chinese youth.

The moderating role of DHL extends the systematic review evidence reported by Xu et al. (12), who found that DHL was positively associated with PA in 80% of examined studies. Our findings add a layer of specificity: DHL does not merely correlate with PA independently but conditions how effectively environmental provisions translate into behavioral engagement. From a social cognitive theory perspective (13), adolescents with greater DHL may possess higher self-efficacy for identifying and utilizing PA resources, interpreting exercise guidelines, and converting school-based PA opportunities into self-initiated activity. This interpretation is consistent with Bandura’s assertion that perceived self-efficacy operates as a cognitive mediator between environmental affordances and behavioral enactment (13, 24).

The urban–rural divergence in the moderation effect warrants particular attention. The pattern whereby DHL amplified the environment-PA pathway in urban but not in rural schools, although the between-group difference in this moderation was not statistically significant, may reflect structural differences in digital infrastructure. Rural adolescents in China continue to face disparities in internet access, digital device availability, and health information quality (4, 15). When the digital ecosystem cannot reliably support health information seeking, even adolescents with nominally high DHL may lack the environmental scaffolding needed to translate literacy into behavior. This interpretation resonates with the scoping review by Li et al. (15), who found that only 2 of 65 PA interventions for Chinese children were conducted in rural regions and that equity considerations were largely absent from intervention design.

### 4.3 Theoretical and practical implications

Theoretically, these findings enrich the social ecological model (5, 6) by demonstrating that an individual-level cognitive resource (DHL) moderates an institutional-level environmental influence on PA. This cross-level interaction supports the model’s core premise that health behavior emerges from the dynamic interplay of factors operating at multiple ecological layers. The results also speak to health equity scholarship (16) by pointing to a potential mechanism (the DHL-environment interaction) through which urban–rural PA disparities may be perpetuated or narrowed; given the cross-sectional design and the statistically inconclusive between-group comparison of the moderation effects, this mechanism should be treated as a candidate pathway requiring longitudinal or experimental confirmation.

Practically, the findings carry three implications for school-based health promotion. First, improving school sports environments, including facility provision, teacher encouragement, and policy enforcement, remains a necessary but insufficient condition for increasing adolescent PA. Second, DHL should be cultivated as a complementary competency: school curricula that integrate digital health information literacy may enhance students’ capacity to leverage environmental PA opportunities. Recent evaluations indicate that brief, self-directed digital resources can indeed improve adolescents’ DHL (25). Third, rural schools require additional digital infrastructure support to ensure that DHL interventions can take effect. Without addressing the digital divide, DHL-focused interventions risk widening rather than narrowing PA disparities. These recommendations align with China’s national policy mandating at least 2 h of comprehensive daily PA for primary and secondary school students and with the WHO Global Action Plan on Physical Activity 2018–2030 (2).

### 4.4 Limitations

Several limitations should be acknowledged. First, the cross-sectional design precludes causal inference. Although the hypothesized directionality (from environment to PA) is theoretically grounded, bidirectional or reciprocal effects cannot be ruled out. Longitudinal or experimental designs are needed to establish temporal precedence. Second, PA was measured via self-report using the IPAQ-SF, which has shown limited criterion validity against accelerometry in Chinese youth (19). Objective PA measurement would strengthen future research. Third, the study was conducted in Hubei Province; while the province includes both urban and rural areas, findings may not generalize to all Chinese regions or to other countries. Fourth, DHL was treated as a unidimensional construct; multidimensional instruments such as the Digital Health Literacy Instrument (26) might reveal more nuanced moderation patterns. Fifth, unmeasured confounders, including family socioeconomic status, peer influence, and neighborhood walkability, may partially account for the observed associations. Sixth, students were nested within 18 schools, and the analyses did not explicitly model this clustered structure. With only 9 urban and 9 rural schools, the effective number of cluster-level units was too small for stable multilevel SEM or cluster-robust estimation (22); consequently, standard errors may be somewhat optimistic, and the large individual-level sample size does not eliminate this concern. Because the primary predictors were student-level perceptions and urban–rural location was modeled through the multigroup design, we expect this limitation to affect the precision of estimates rather than the substantive pattern of findings, but studies with larger numbers of clusters should employ multilevel modeling. Seventh, the three IPAQ-SF intensity scores were modeled as reflective indicators of a single latent PA construct; although the sensitivity analysis with observed total log-MET scores reproduced the main conclusions, formative or single-indicator specifications may be more appropriate for behavioral measures of this kind.

### 4.5 Future directions

Future research should pursue several extensions. Longitudinal designs with multiple assessment waves would enable examination of whether DHL and school environment interact over time to shape PA trajectories. Intervention studies that experimentally manipulate DHL (e.g., through digital health literacy curricula) (25) within schools of varying environmental quality would provide causal evidence. Research incorporating objective PA measurement and multi-level modeling that nests students within schools would further strengthen inference. Finally, qualitative inquiry into how rural adolescents navigate digital health information in resource-constrained settings could illuminate the mechanisms behind the absent moderation effect observed in rural schools.

## 5 Conclusion

This study demonstrates that digital health literacy moderates the relationship between school sports environment and adolescent PA. The moderation was statistically significant in urban but not rural schools, although the between-group difference in the moderation effect was not statistically significant; the environment-PA association itself was significantly stronger in urban schools. These patterns point to a potential pathway through which health inequities may be sustained, one that requires longitudinal confirmation. School-based health promotion policies that simultaneously improve sports environments, cultivate digital health literacy, and narrow the digital divide are more likely to achieve equitable PA outcomes for all adolescents. These evidence-based strategies directly contribute to the public health objectives articulated in Sustainable Development Goals 3 and 10 and to China’s national target of ensuring comprehensive daily PA for all students.

## Data Availability

The raw data supporting the conclusions of this article will be made available by the authors, without undue reservation.
